# Amelioration of Mouse Retinal Degeneration After Blue LED Exposure by Glycyrrhizic Acid-Mediated Inhibition of Inflammation

**DOI:** 10.3389/fncel.2019.00319

**Published:** 2019-07-16

**Authors:** Gyu Hyun Kim, Sun-Sook Paik, Yong Soo Park, Hyoun Geun Kim, In-Beom Kim

**Affiliations:** ^1^Department of Anatomy, College of Medicine, The Catholic University of Korea, Seoul, South Korea; ^2^Catholic Institute for Applied Anatomy, College of Medicine, The Catholic University of Korea, Seoul, South Korea

**Keywords:** glycyrrhizic acid, HMGB1, inflammation, photoreceptor, retinal degeneration

## Abstract

Glycyrrhizic acid (GA) is a major component in the root and rhizomes of licorice (*Glycyrrhiza glabra*), which have been used as an herbal medicine, because of its anti-inflammatory activity. GA is known as an inhibitor of high-mobility group box 1 (HMGB1), which is involved in the pathogenesis of various inflammatory diseases including inner retinal neuropathy. In this study, we examined the effect of GA in a mouse model of retinal degeneration (RD), the leading cause of blindness. RD was induced by exposure to a blue light-emitting diode (LED). In functional assessment, electroretinography showed that the amplitudes of both a- and b-waves were reduced in RD mice, whereas they were significantly increased in GA-treated RD mice (*P* < 0.05), compared to those in non-treated RD animals. In histological assessment, GA treatment preserved the outer nuclear layer where photoreceptors reside and reduced photoreceptor cell death. GA-treated retinas showed significantly reduced expression of proinflammatory cytokines such as TNF-α, IL-6, IL-1β, CCL2 and 6, iNOS, and COX-2 (*P* < 0.05), compared to that in non-treated retinas. Immunohistochemistry showed that Iba-1 and GFAP expression was markedly reduced in GA-treated retinas, indicating decreased glial response and inflammation. Interestingly, HMGB1 expression was reduced in non-treated RD retinas whereas GA paradoxically increased its expression. These results demonstrate that GA preserves retinal structure and function by inhibiting inflammation in blue LED-induced RD, suggesting a potential application of GA as a medication for RD. In addition, we propose a potential retinal protective function of HMGB1 in the pathogenesis of RD.

## Introduction

Retinal degeneration (RD) is a heterogeneous group of diseases characterized by the irreversible and progressive degeneration of photoreceptor cells in the retina, leading to blindness (Papermaster and Windle, 1995; Gregory-Evans and Bhattacharya, 1998; Kim G.H. et al., 2016). In the pathogenesis of a representative RD type of age-related macular degeneration (AMD) resulting from aging and long-term light exposure, inflammation is thought to be critical (Coleman et al., 2008; Ding et al., 2009; Karlstetter et al., 2010; Kim G.H. et al., 2016). Microglia and macrophages are believed to play an important role in the initiation and propagation of inflammatory responses and subsequent neuronal cell death in AMD (Karlstetter et al., 2010; Madeira et al., 2015) and in light-induced RD models (Chang et al., 2016; Kim G.H. et al., 2016).

Glycyrrhizic acid (GA) is a major sweet-tasting component of licorice (*Glycyrrhiza glabra*) root, which has been used in herbalism and oriental traditional medicine due to its natural anti-inflammatory effects (Kim et al., 2012; Ming and Yin, 2013; Kao et al., 2014). Its main mechanism involves inhibition of high mobility group box 1 (HMGB1), which has chemoattractant, mitogenic, and cytokine-like activities, *via* its direct binding (Mollica et al., 2007; Kim et al., 2012; Shen et al., 2015). In the retina, GA has inhibitory effects on inner retinal neuropathies, such as diabetic retinopathy (Chen et al., 2013; Abu El-Asrar et al., 2014), NMDA-induced injury (Sakamoto et al., 2015; Sakamoto et al., 2017), and ischemia-reperfusion injury (Dvoriantchikova et al., 2011; Liu et al., 2017), in which amacrine and ganglion cells die. In addition, increased level of HMGB1 has been found in a rat retinal detachment model and in human eyes with retinal detachment (Arimura et al., 2009), in which photoreceptors mainly die. However, the therapeutic potential of GA in retinal detachment has not been tested. Thus, the inhibitory effects of GA on outer retinal neuropathy and RD remain unclear.

Therefore, in the present study, we investigated whether GA has inhibitory effects on massive photoreceptor cell death in RD induced by blue light-emitting diode (LED) exposure in mice *via* anti-inflammation.

## Materials and Methods

### Animals

A total of 39 male albino BALB/c mice, 7 weeks of age, were used in this study. Twenty-one mice (*n* = 3, each group) were employed for the screening of GA effect and determination of an appropriate concentration of GA; 18 mice (*n* = 6, each group) were used for the main study. They were kept in a plastic cage in a climate-controlled laboratory with a 12 h (7 a.m. to 7 p.m.) light/dark cycle. The animals were randomly assigned to the treatment group. All mice-related experiments were handled according to the regulations of the Catholic Ethics Committee of the Catholic University of Korea, Seoul, which conform to the National Institute of Health (NIH) guidelines for the Care and Use of Laboratory Animals (NIH Publication No. 80-23), as revised in 1996. Experimental procedures were approved by the Institutional Animal Care and Use Committee at the College of Medicine, The Catholic University of Korea (Approval Numbers: CUMC 2016-0172-12 and 2017-0241-02).

### Exposure to Blue LED

As described previously (Kim G.H. et al., 2016), BALB/c mice were dark adapted for 24 h and their pupils were then dilated with 0.5% tropicamide and 0.5% phenylephrine hydrochloride eye drops (Santen, Osaka, Japan) 30 min before exposure to a blue LED. Non-anesthetized mice were then exposed to 2000 lux of blue LED (460 ± 10 nm) for 2 h in cages with reflective interiors. Light intensities were measured using an LED light meter (model TM-201L, TENMARS Electronics, Taipei, Taiwan). After exposure to blue LED, animals were kept in darkness for 24 h, and then resumed a 12 h light-dark cycle for 3 days.

### Administration of GA

Glycyrrhizic acid was obtained from Sigma-Aldrich Corp., (St. Louis, MO, United States). GA in distilled water (DW) at a dose of 1, 2.5, 5, 10, and 20 mg/kg or an identical volume of DW was injected intravenously *via* the tail vein 30 min before exposure to blue LED.

### Electroretinography (ERG)

Electroretinography (ERG) recordings followed procedures described in our previous study (Kim G.H. et al., 2016). In brief, the mice were kept in a completely dark room for 16 h before the ERG recording. All animals were anesthetized intraperitoneally with zolazepam (20 mg/kg) and xylazine (7.5 mg/kg). The corneas were coated with hydroxypropyl methylcellulose gel and covered with gold ring contact electrodes. A ground electrode and reference electrode were placed subcutaneously in the tail and ear, respectively. Stimuli were brief white flashes delivered *via* a Ganzfeld stimulator (UTAS-3000; LKC Technologies, Gaithersburg, MD, United States). Signals were amplified and filtered through a digital band-pass filter ranging from 5 to 300 Hz to yield a- and b-waves. Scotopic ERG, rod-mediated responses were obtained at the following increasing light intensities: 0.025 and 3.96 cd/s⋅m^2^. Photopic, cone-mediated responses were obtained following 5 min light adaptation on the background light intensity. Recordings were obtained at the light intensity of 6.28 cd/s⋅m^2^. Each record was the average of three responses obtained within a 15-seconds inter-stimulus interval. The amplitude of the a-wave was measured from the baseline to the maximum a-wave peak, and the b-wave was measured from the maximum a-wave peak to the maximum b-wave peak.

### Histological Analysis

At 3 days after blue LED exposure, the eye cup was enucleated and fixed in 4% paraformaldehyde for 2 h. After fixation, the eye cup was rinsed in 0.1 M phosphate buffer (PB; pH 7.4), transferred to 30% sucrose, infiltrated overnight, and embedded in a supporting medium for frozen tissue specimens (Tissue-Tek O.C.T. compound; Sakura Finetek, Alphen aan den Rijn, Netherlands). As described in our previous report (Kim G.H. et al., 2016), retinal sections (7-μm in thickness) were cut in the sagittal plane, and stained with hematoxylin and eosin (H&E). Quantitative analysis was performed in the stained sections; outer nuclear layer (ONL) thicknesses were measured at 240-μm intervals (superior to inferior) on vertical strips of the retina.

### Terminal Deoxynucleotidyl Transferase dUTP Nick End Labeling (TUNEL) Assay

TUNEL assays were performed in accordance with the manufacturer’s protocols (In Situ Cell Death Detection kit; Roche Biochemical, Mannheim, Germany) to detect retinal cell death. In cryo-sections of the eye cup preparations, cell nuclei were counterstained with 4′,6-diamidino-2-phenylindole (DAPI; dilution, 1:1000; Invitrogen, Eugene, OR, United States). Light microscopic images were acquired on a Zeiss LSM 510 Meta confocal microscope (Carl Zeiss Co., Ltd., Oberkochen, Germany). The number of TUNEL-positive cells in the ONL was counted in two sections at a distance between 480 and 720 μm from the optic nerve in the superior area of the retina.

### Immunohistochemistry

After washing with 0.01 M PBS, retinal sections were pre-incubated in 10% normal donkey serum for 1 h at room temperature. The sections were then incubated with rabbit polyclonal anti-ionized calcium binding adaptor molecule 1 (Iba-1) antibody (1:1000; Wako Pure Chemical Industries, Osaka, Japan), rabbit polyclonal anti-glial fibrillary acidic protein (GFAP) antibody (1:1000; Chemicon, Temecula, CA, United States), or rabbit polyclonal anti-HMGB1 antibody (dilution, 1:000; Abcam, Cambridge, MA, United States) diluted in PBS, for 1 d at 4°C. Sections were subsequently washed in PBS and incubated with Cy3-conjugated (dilution, 1:3000; Jackson ImmunoResearch, West Grove, PA, United States) or Alexa 488-conjugated donkey anti-rabbit IgG (1:3000; Molecular Probes, Eugene, OR, United States) for 2 h at room temperature. After rinsing several times in PBS, the cell nuclei were fluorescent specimens were counterstained with DAPI for 10 min and then mounted with anti-fading mounting media (Vector Laboratories, Burlingame, CA, United States). Images were obtained using a Zeiss LSM 510 Meta confocal microscope (Carl Zeiss Co., Ltd.). Quantitative image analysis was performed using Zen 2.3 software (Blue edition; Carl Zeiss). Region of interest was selected at a distance of 300 μm from the optic disk of each retinal section, and intensity of Iba-1 and GFAP immunoreactivities was automatically measured.

### Quantitative Real-Time PCR (Real-Time qPCR)

Total RNA was purified with easy-Blue reagent (iNtRON Bio) according to the manufacturer’s instructions. First-strand cDNA was synthesized with reverse transcriptase using the PrimeScript RT reagent kit (Takara Bio, Japan) in a total volume of 10 μL containing 0.5 μg of total RNA.

Real-time qPCR was performed in a final volume of 20 μL containing 10 μL of 2× SYBR Premix Ex Taq (Takara Bio, Japan), 1 μL each of 10 pmol/μL forward and reverse primers, and 2 μL cDNA template (1/100 dilution), using a commercial PCR detection system (LightCycler^®^ 480, Roche, Mannheim, Germany) following the manufacturer’s instructions. The annealing temperature was increased to 56°C for amplification. Melting curve analysis confirmed that each product was homogeneous and specific. Relative expression was calculated by comparison with a standard curve after normalization to the expression of the housekeeping gene GAPDH chosen as the control. The following primer sets were used: 5′-CCTTGTCTACTCCCA GGTTC-3′ (forward) and 5′-AGGAGGTTGACTTTCTCCTG-3′ (reverse) for TNF-α; 5′-TGTTCAAAGAGAGCC TGTGT-3′ (forward) and 5′-ATGTCCCCTTGAATCAACTT-3′ (reverse) for IL-1β; 5′-CCATCCAATTCATCTTGAAA-3′ (forward) and 5′-GAGGAATGTCCACAAACTGA-3′ (reverse) for IL-6; 5′-GCTACTCATTCACCAGCAAG-3′ (forward) and 5′-TGAGCTTGGTGACAAAAACT-3′ (reverse)for CCL2 for CCL2; 5′-TGTTTGTCACTCGAAGGAAC-3′ (forward) and 5′-AGGGTCAGAATCAAGAAACC-3′ (reverse) for CCL5; 5′- -3′ (forward) and 5′- -3′ (reverse) for; 5′-TGAAAGTGGTGTTCT TTGCT-3′ (forward) and 5′-TGGCTAGTGCTTCAGACTTC-3′ (reverse) for iNOS; 5′-AAAAATGCTGCAGGTATCAA-3′ (forward) and 5′-ACCCCTTTGTTTGATGAGAT-3′ (reverse) for COX-2; 5′-TGTATGTATGGGGAGAGCTG-3′ (forward) and 5′-TTCACCACCTTCTTGATGTC-3′ (reverse) for GAPDH. Real-time qPCR was performed three times for each group.

### Western Blotting

Western blot analyses were performed on retinal extracts of the retina, which were homogenized in ice-cold lysis buffer (1% sodium dodecyl sulfate, 1.0 mM sodium orthovanadate, 10 mM Tris, pH 7.4). Aliquots of lysed tissue, each containing 50 μg of total protein were heated at 100°C for 10 min with an equivalent volume of 2× sample buffer and were loaded onto 10% polyacrylamide gels. Proteins were electrophoresed and subsequently blotted onto a polyvinylidene fluoride membrane. The membrane was blocked with 5% non-fat dry milk dissolved in 0.01 M PBS (pH 7.4) containing 0.05% Tween-20 for 1 h at room temperature. The membrane was then incubated for 15 h at 4°C with rabbit anti-HMGB1 polyclonal antibody (1:1000; Abcam) in blocking solution. The membrane was rinsed 3 times with PBS containing 0.05% Tween-20 (10 min per wash), and was then incubated with peroxidase-conjugated donkey anti-goat IgG antibody (1:1000; Jackson ImmunoResearch) for 2 h at room temperature. Blots were developed using the Enhanced Chemiluminescence Detection Kit (Amersham, Arlington Heights, IL, United States) and densitometry was performed using the Eagle Eye TMII Still Video System (Stratagene, La Jolla, CA, United States).

### Statistical Analysis

Data are presented as mean ± SEM. All statistical analyses for ERG amplitude, histology image analysis, TUNEL-positive quantification, immunohistochemistry, real-time qPCR, and western blot analyses were conducted in Graphpad Prism 5.0 (GraphPad Software, San Diego, CA, United States) by one-way ANOVA with Bonferroni’s *post hoc* test comparing the mean of each group with the mean of every other group. For all tests, the differences were considered statistically significant at *P* < 0.05.

## Results

### Determination of GA Dosage

**FIGURE 1:**
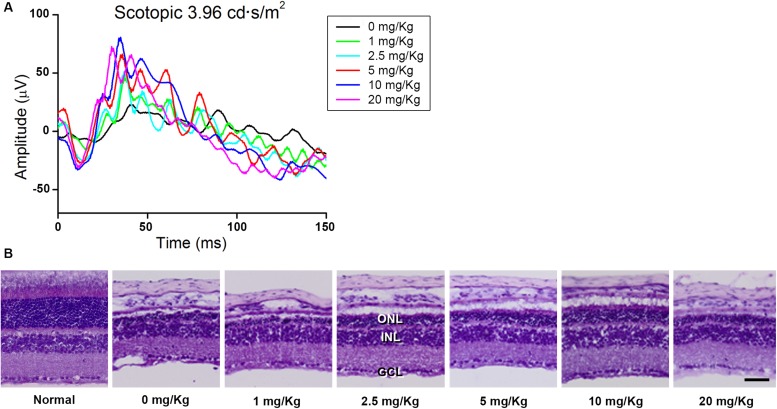
Dose-dependent protective effect of GA against blue LED-induced RD. **(A)** ERG analysis. The amplitudes of both a- and b-waves of the ERG responses increased in a dose-dependent manner. **(B)** H&E staining. Retinal structure was better preserved in the group treated with 10 mg/kg GA than in other groups treated with various concentrations of GA. Scale bar, 50 μm.

First, we screened the inhibitory effect of GA on blue LED-induced RD and determined the appropriate dose of GA. For this purpose, we performed scotopic ERG on the mouse eye at 3 days after blue LED exposure and assessed the histology in retinal sections stained with H&E after ERG recording (*n* = 3, each group). As shown in Figure 1, GA had an inhibitory effect on RD histologically and functionally in a dose-dependent manner. The best dose for administration in this study was identified as 10 mg/kg. In the group of 20 mg/kg, its inhibitory effect was decreased.

### GA Preserves Retinal Function in Blue LED-Induced RD

**FIGURE 2:**
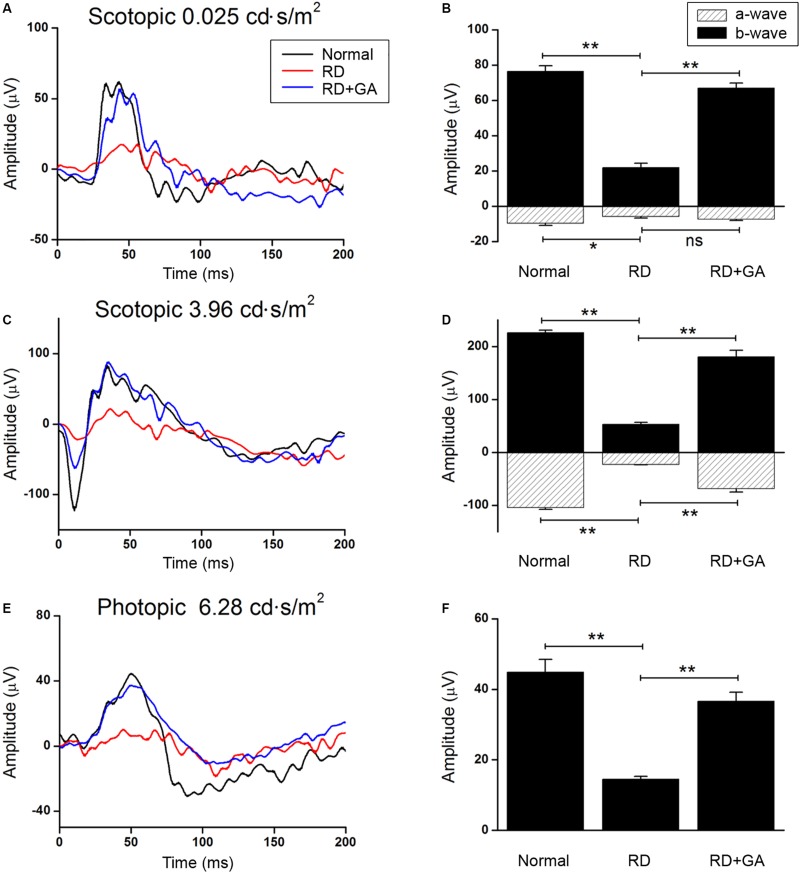
ERG recordings to evaluate the functional effect of GA on blue LED-induced RD. **(A–D)** Scotopic ERG recordings at 0.025 **(A)** and 3.96 cd.s/m^2^. **(C)** Light intensities were obtained from the normal control mice (dark curve), untreated control RD mice (red curve), and GA-treated RD mice (blue curve), respectively. Quantitative analyses of the a- and b-wave amplitudes of the ERGs were denoted with filled- and dashed-columns **(B,D)**. **(E)** Photopic ERG recordings at 6.28 cd.s/m^2^ light intensity were carried out from the normal control, RD control, and GA-treated RD mice. **(F)** Quantitative analysis of b-wave amplitudes of the photopic ERG results was summarized in a bar graph. The data are shown as the mean ± SEM; *n* = 6, ^*^P < 0.05, ^∗∗^*P* < 0.01.

We evaluated the functional effect of GA against RD with ERG in detail. Figure 2 shows the representative scotopic and photopic ERG recordings in age-matched normal control mice, DW-treated RD control, and GA-treated RD mice at 3 days after blue LED exposure, under different light intensities. The flash intensity of 0.025 cd.s/m^2^ was found to be the lowest scotopic level to yield the a-wave, with a reliable b-wave (Figures 2A,B) and the optimal luminance to produce both ERG components with maximal amplitudes was about 3.96 cd.s/m^2^ (Figures 2C,D). In this scotopic condition (Figures 2C,D), a- and b-wave amplitudes in GA-treated RD mice (*n* = 6, a-wave: 68.6 ± 6.6 μV, b-wave: 180.8 ± 12.2 μV) were almost comparable to those in the normal control mice (*n* = 6, a-wave: 104.2 ± 3.4 μV, b-wave: 226.3 ± 4.8 μV) and were significantly higher than those in RD mice (*n* = 6, a-wave: 22.9 ± 0.7 μV, b-wave: 53.1 ± 3.2 μV) (*P* < 0.05). The differences in amplitudes of a- and b-waves between RD and GA-treated RD mice were 299% (22.9 ± 0.7 μV vs. 68.6 ± 6.6 μV) and 340% (53.1 ± 3.2 μV vs. 180.8 ± 12.2 μV), respectively. The photopic ERG recordings are represented in Figures 2E,F at a flash intensity of 6.28 cd.s/m^2^, which evoked b-waves (Figures 2E,F) without a-waves. Under the photopic condition, ERG responses in mice groups were almost similar to the scotopic responses, which show reduction and enhancement of the amplitudes in RD mice and in GA-treated RD mice, respectively. Figure 2F shows that b-wave amplitudes in GA-treated RD mice are about 80% (*n* = 6, 36.5 ± 0.8 μV) of those in the normal control mice (*n* = 6, 45.6 ± 3.5 μV) and are significantly higher than those in RD mice (*n* = 6, 14.6 ± 2.5 μV) (*P* < 0.05).

### GA Preserves Retinal Histology in Blue LED-Induced RD *via* Inhibition of Photoreceptor Cell Death

**FIGURE 3:**
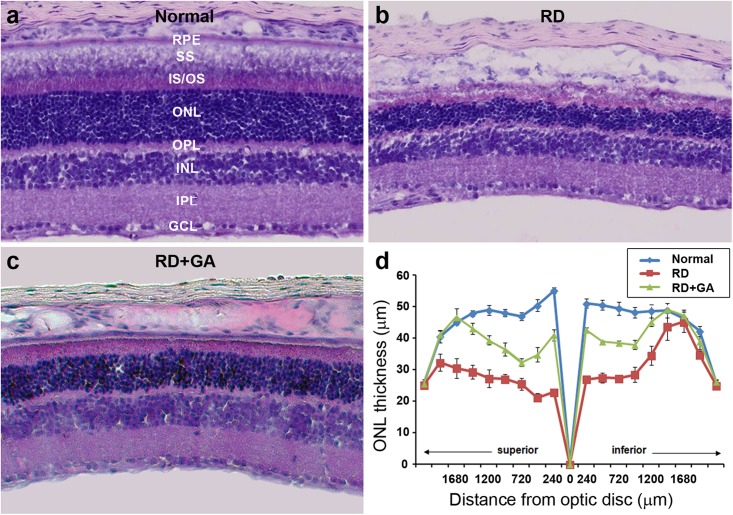
Histological analysis to evaluate the structural effect of GA on blue LED-induced RD. **(a–c)** Retinal cross-sections of normal control **(a)**, untreated RD control **(b)**, and GA-treated RD mice **(c)** were obtained at 3 days after RD. Scale bar, 100 μm. **(d)** The thickness of the outer nuclear layer was measured at every 240 μm from the optic nerve. Data are shown as mean ± SEM; *n* = 5.

After ERG recordings, histological analysis was performed. Although laminar structure of the retina was generally preserved in RD control and GA-treated RD mice, two prominent changes were easily observed in the ONL where photoreceptors are located (Figure 3). The most prominent was the change in the ONL thickness. That is, at 480 μm from the optic disk in the inferior retina, the thickness of the ONL in the RD control and GA-treated RD mice corresponded to 54 and 77%, compared to that in normal control mice (Figures 3a–d). The other change was the arrangement of the ONL. In normal control mice, the two borderlines of the ONL appeared to be clean lines (Figure 3a), whereas those in RD mice were much wobbly (Figure 3b). In GA-treated RD mice, both lines appeared to be modest (Figure 3c).

**FIGURE 4:**
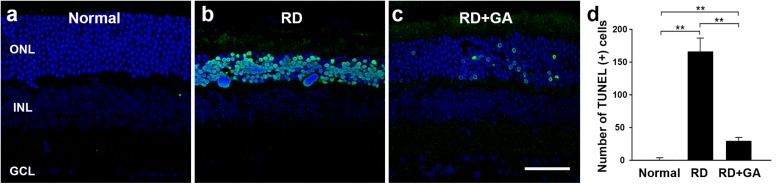
TUNEL analysis to evaluate the photoreceptor protective effect of GA on blue LED-induced RD. **(a–c)** TUNEL-positive photoreceptors (green) in the outer nuclear layer were much more observed in the untreated control RD mouse retina **(b)** at 3 days after RD than those in the normal control mouse retina **(a)**. A lower number of TUNEL-positive photoreceptors was observed in GA-treated RD mouse retina **(c)**, compared to that in the untreated RD mouse retina **(b)**. Scale bar, 50 μm. **(d)** Quantitative analysis of the number of TUNEL-positive photoreceptors was conducted. Data are shown as mean ± SEM; *n* = 5, ^∗∗^*P* < 0.01.

Next, we evaluated the GA effects on photoreceptor cell death using the TUNEL assay (Figure 4). As previously reported (Kim G.H. et al., 2016), none or few TUNEL labeling was seen in the ONL (Figure 4a), while numerous TUNEL-positive photoreceptors were identified throughout the ONL in RD mice (Figure 4b). However, in GA-treated RD mice, TUNEL-positive photoreceptors were significantly reduced (Figure 4c). Quantification (*n* = 5 in each group) confirms and demonstrates a significant reduction in TUNEL-positive photoreceptors (*P* < 0.01) (Figure 4d). These results suggest that GA inhibits photoreceptor cell death and preserves the structure and function of the retina in blue LED-induced RD.

### GA Inhibits Inflammation in Blue LED-Induced RD

**FIGURE 5:**
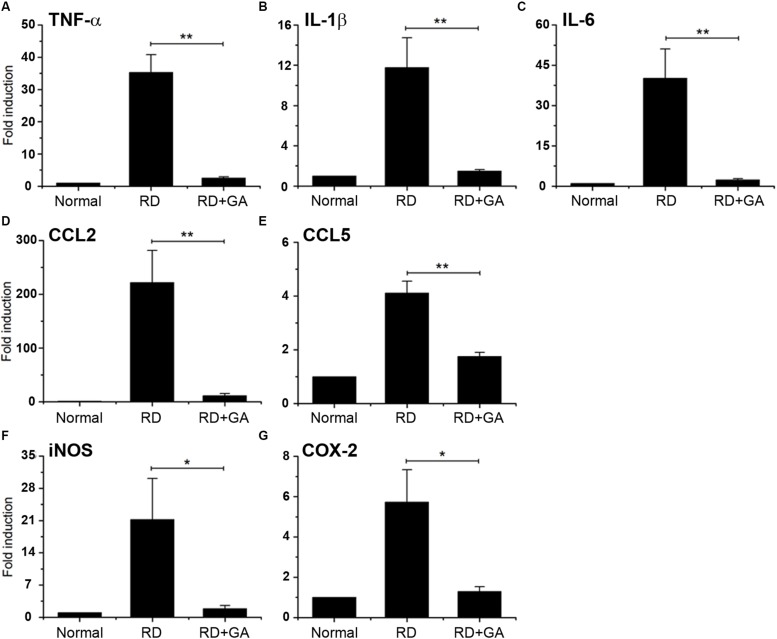
Real-time qPCR analysis to evaluate the anti-inflammatory effect of GA on blue LED-induced RD. **(A–G)** mRNA expression levels of TNFα **(A)**, IL-6 **(B)**, IL-1β **(C)**, CCL2 **(D)**, CCL5 **(E)**, iNOS **(F)**, and Cox-2 **(G)** in GA-treated RD retinas at 3 days after RD were significantly reduced, compared to those in the untreated RD retinas. Data are shown as mean ± SEM; *n* = 5, ^*^P < 0.05, ^∗∗^*P* < 0.01.

We assessed inflammation in blue LED-induced RD and the anti-inflammatory effects of GA against RD. Analysis of gene expression by real-time qPCR showed the differential expression of several genes related to inflammation in normal control, RD control, and GA-treated RD mice (Figure 5). In the RNA isolated from the RD mice, the following seven genes of inflammatory mediators were upregulated, compared to the normal controls (Figure 5): three proinflammatory cytokines, TNF-α (*P* < 0.01), IL-1β (*P* < 0.01), and IL-6 (*P* < 0.01); two chemokines, CCL2 (*P* < 0.01) and CCL5 (*P* < 0.01); two other inflammatory mediators, iNOS (*P* < 0.05) and COX-2 (*P* < 0.05). In GA-treated RD mice, all genes were significantly downregulated compared to those in the RD controls (^*^P < 0.05, ^∗∗^*P* < 0.01 in Figure 5), and were thus comparable to the normal controls (Figure 5). Therefore, these results demonstrate that GA preserves the retina by inhibiting inflammation in LED-induced RD.

### GA Inhibits Glial Responses in Blue LED-Induced RD

**FIGURE 6:**
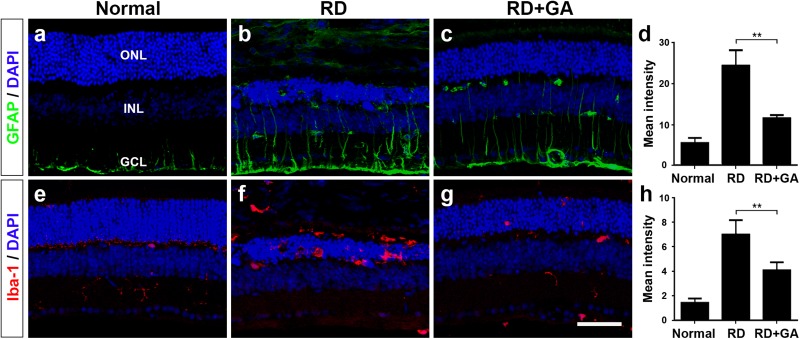
Immunohistochemistry and image analyses to evaluate glial responses of GA on blue LED-induced RD. **(a–h)** Confocal micrographs from vertical sections of normal **(a,e)**, untreated RD control **(b,f)**, and GA-treated RD retinas **(c,g)** processed for GFAP (green in **a–c**) and Iba-1 (red in **e–g**) immunoreactivities. Quantitative image analyses of the immunoreactivities for GFAP **(d)** and Iba-1 **(h)** were conducted. Both GFAP and Iba-1 immunoreactivities were significantly reduced in the retinas of the GA-treated group, compared with the untreated RD group. Scale bar, 50 μm. Data are shown as mean ± SEM; *n* = 5, ^∗∗^*P* < 0.01.

As previously reported (Chang et al., 2016; Kim G.H. et al., 2016), two retinal glial cells, Müller cells and microglia are activated in blue LED-induced RD. We assessed the effects of GA on glial responses using immunohistochemistry with anti-GFAP, a marker of activated Müller cells, and anti-Iba-1, a microglial marker (Figure 6). In normal control retina, GFAP was expressed in the endfeet and proximal processes of Müller cells in the ganglion cell layer (GCL) and the inner plexiform layer (IPL) (Figure 6a). The expression level of GFAP in control RD mice was higher than that in normal control mice; thus, GFAP immunoreactivity was frequently seen in the inner nuclear layer (INL), as well as in the GCL and the IPL (Figure 6b), and infrequently in the ONL. However, GFAP expression in GA-treated RD mice was significantly decreased (*P* < 0.05) and thus, appeared to be similar to that in normal control mice (Figures 6c,d).

Iba-1 expression patterns were quite similar to GFAP expression patterns. That is, Iba-1 was weakly expressed in microglia in the IPL in the normal control (Figure 6e), whereas it was strongly expressed in microglia in the ONL and in the subretinal space in RD (Figure 6f). In GA-treated RD mice, Iba-1 expression in microglia in the ONL and in subretinal space was significantly decreased (*P* < 0.05) and thus, rarely observed (Figures 6g,h). Taken together, GA might have strong inhibitory effects on retinal glial responses in blue LED-induced RD.

### GA Increases HMGB1 Expression Level by Inhibition of HMGB1 Release From Photoreceptors During RD

**FIGURE 7:**
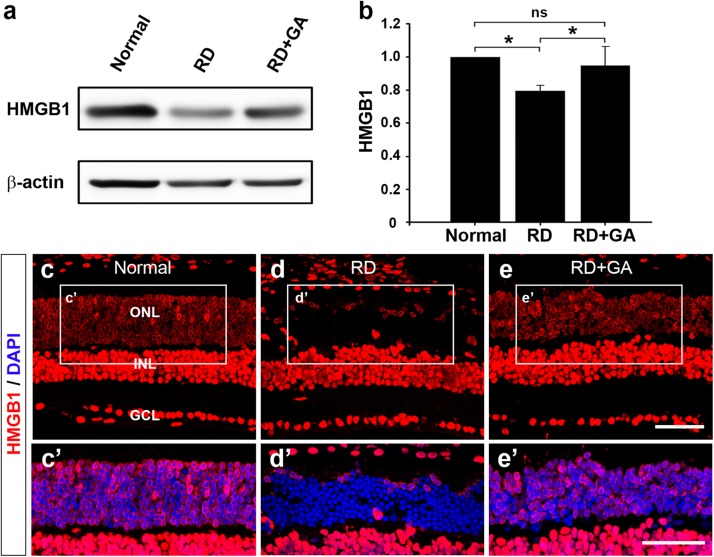
The effect of GA on HMGB1 expression in RD retinas at 3 days after RD. **(a,b)** Western blot analysis. A ∼29 kDa band is recognized by anti-HMGB1 in each sample **(a)** and densitometric analysis of the band is represented **(b)**. The intensity of band was normalized to the normal as fold change. Data represent the mean ± SD (*n* = 6, each group). ^*^P < 0.05; ns, not significant. **(c,e)** Confocal micrographs from vertical sections of normal **(c)**, untreated RD control **(d)** and GA-treated RD retinas **(e)** processed for HMGB1 (red). Higher-magnification views of the rectangles in panels **(a–c)**, focusing on the outer nuclear layer (ONL) with DAPI-counterstained photoreceptors, show changes in HMGB1 distribution and cellular localization in untreated RD control **(d’)** and GA-treated RD retinas **(e’)**, compared to the normal retina **(c’)**. Scale bars, 50 μm.

As the main mechanism of GA against inflammation involves inhibition of HMGB1 (Mollica et al., 2007; Kim et al., 2012; Shen et al., 2015), we first examined the changes in HMGB1 levels by western blot analysis (Figures 7a,b). The analysis demonstrated that HMGB1 expression in the RD retinas was significantly decreased compared to that in normal controls (*P* < 0.05), whereas that in GA-treated RD retinas was significantly increased compared to that in RD controls (*P* < 0.05) and was thus similar to that in normal controls (Figures 7a,b).

We also performed immunohistochemistry with anti-HMGB1 to examine changes in HMGB1 expression (Figures 7c–e). In the retina of normal control mice, HMGB1 was expressed in all retinal neurons (Figure 7c’). Consistent with a previous report (Hoppe et al., 2007), HMGB1 immunoreactivity was found homogeneously in the somata of the bipolar, amacrine, ganglion cells, and in the retinal pigment epithelium, whereas it was observed in periphery of somata in the photoreceptors. In the retina of RD mice, HMGB1 immunoreactivity in most photoreceptors that were localized in the ONL disappeared (Figure 7d’). In GA-treated RD retinas, the pattern of HMGB1 immunoreactivity changed in the RD retinas and was restored to that in the normal control retinas (Figure 7e’). These results indicate that HMGB1 expression is decreased in the retina in RD whereas GA attenuates HMGB1 release from the photoreceptors during RD.

## Discussion

There have been growing evidences that inflammation is an important event in the pathogenesis of RD. Recent clinical reports have shown that inflammatory reactions underlie AMD (Coleman et al., 2008; Nita et al., 2014; Kauppinen et al., 2016), retinitis pigmentosa (RP) (Yoshida et al., 2013; McMurtrey and Tso, 2018), and retinal detachment (Arimura et al., 2009; Murakami et al., 2013), and that anti-inflammatory agents can ameliorate them (Coleman et al., 2008; Becerra et al., 2011; Viringipurampeer et al., 2013; Wubben et al., 2016; Bandello et al., 2017). Previously, we introduced the blue LED-induced RD mouse model and demonstrated its inflammatory characters: advent and activation of microglial cells in the ONL and increased expression of retinal inflammation markers, such as GFAP and osteopontin (Chang et al., 2016; Kim G.H. et al., 2016). In this study, we demonstrated that expression of proinflammatory cytokines, such as TNF-α, IL-1β, and IL-6, chemokines, such as CCL2 and CCL5, and other inflammatory mediators, such as iNOS and COX-2, was significantly increased. These inflammatory signs have been reduced by administration of GA, which has an anti-inflammatory effect. These results corroborate the concept that inflammation is a critical event in the pathogenesis of RD, and indicate that the blue LED-induced RD model is a useful model to study RD.

In RD, apoptosis has been considered the main mechanism of photoreceptor cell death (AMD (Xu et al., 1996; Dunaief et al., 2002), RP (Portera-Cailliau et al., 1994; Cottet and Schorderet, 2009), and retinal detachment (Cook et al., 1995; Arroyo et al., 2005), and thus, many pharmacological trials targeting anti-apoptotic molecules have been conducted. Unfortunately, the trials have not produced significant therapeutic effects (Donovan and Cotter, 2002; Murakami et al., 2013; Chinskey et al., 2014; Guadagni et al., 2015). A recently growing body of studies has shown that photoreceptor cell death in RD involves necrosis and autophagy as well as apoptosis (Sancho-Pelluz et al., 2008; Murakami et al., 2013; Chinskey et al., 2014; Guadagni et al., 2015; Lin and Xu, 2019). Previously, we have also demonstrated that the blue LED-induced RD model used in this study showed characters of necrotic photoreceptor cell death and that a necrosis inhibitor, NecroX-5 significantly prevented photoreceptor degeneration (Kim H.I. et al., 2016). In addition, necroptosis, a programmed necrosis pathway, is recently in the limelight as an important mechanism in RD pathogenesis. In a variety of RD models, photoreceptor cell death was mediated by receptor interacting protein (RIP) kinases, RIP1/RIP3 kinases (AMD (Murakami et al., 2014), RP (Murakami et al., 2012; Sato et al., 2013), and retinal detachment (Trichonas et al., 2010).

High-mobility group box 1 is a highly conserved chromatin binding protein and regulates gene expression and nucleosome stability. It also functions as a damage-associated molecular pattern (DAMP) molecule that can be passively released by necrotic cells and actively secreted by inflammatory cells such as macrophages and monocytes under various pathological conditions (Scaffidi et al., 2002; Lotze and Tracey, 2005; Klune et al., 2008). In experimental and clinical RD, HMGB1 released from necrotic and/or necroptotic photoreceptors is reported to trigger and progress retinal inflammation, and thus deteriorate RD (Arimura et al., 2009; Murakami et al., 2014; Allocca et al., 2019). Thus, we hypothesized that HMGB1 is a key molecule in RD pathogenesis and is a potent molecular target for inhibition of RD, and thus tested whether GA, an HMGB1 inhibitor, can effectively inhibit RD in a blue LED-induced RD model.

**FIGURE 8:**
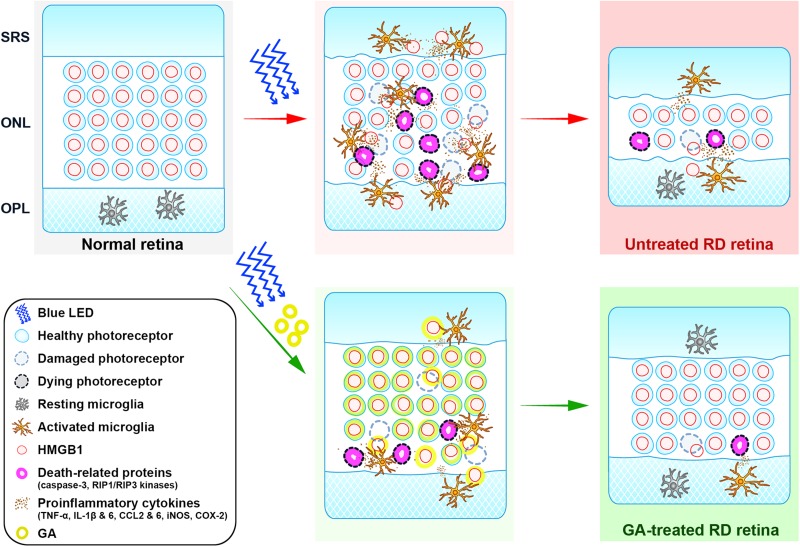
Cartoon summary. The diagram illustrates the role of HMGB1 in the pathogenesis of blue-LED induced RD and amelioration of RD by GA. HMGB1 released from damaged photoreceptors by blue-LED exposure acts as a damp, which activates microglia. Activated microglia release proinflammatory cytokines such as TNF-α, IL-6, IL-1β, CCL2 and 6, iNOS, and COX-2, which lead to the enhancement of inflammation or cell-death signaling in photoreceptors, such as caspase-3 and RIP1/RIP3 kinases. In this pathologic process, GA acts in two modes: (1) the classical mode is to inhibit retinal inflammation by binding to released HMGB1, thereby inhibiting the activation of microglia and proinflammatory cytokine release; (2) the photoreceptor-specific mode is to protect photoreceptors by binding to nuclear HMGB1 in photoreceptors, thus leading to the stabilization of the genome against injury or inhibition of the expression of death-related genes. ONL, outer nuclear layer; OPL, outer plexiform layer; SRS, subretinal space.

Glycyrrhizic acid binds directly to HMGB1 by interacting with two shallow concave surfaces formed by the two arms of both HMG boxes (Sakamoto et al., 2015; Sakamoto et al., 2017). Further, binding of HMGB1 and GA has been shown to inhibit the chemoattractant, mitogenic, and cytokine-like activities of HMGB1, leading to anti-inflammatory effects (Mollica et al., 2007). These characters make HMGB1 a critical molecular target in a variety of human diseases, and thus, GA as a pharmacological strategy to inhibit HMGB1 has been used both *in vivo* and *in vitro* (Ming and Yin, 2013; Kang et al., 2014; Kao et al., 2014). In retinal diseases, it has been demonstrated that GA has inhibitory effects on inner retinal neuropathy, such as diabetic retinopathy (Chen et al., 2013; Abu El-Asrar et al., 2014), NMDA-induced injury (Sakamoto et al., 2015; Sakamoto et al., 2017), and ischemia-reperfusion injury (Dvoriantchikova et al., 2011; Liu et al., 2017), in which amacrine and ganglion cells die. In addition, RD models with photoreceptor death are reported to show increased levels of HMGB1 in a rat retinal detachment model and in human eyes with retinal detachment (Viringipurampeer et al., 2013) as well as in an RD model induced by dsRNA injection (Murakami et al., 2014). *Rip3*^–/–^ mice, in which the necroptotic cell death pathway was blocked, were shown to attenuate HMGB1 release and finally lead to RD inhibition (Allocca et al., 2019). In this study, we demonstrated that GA significantly reduces photoreceptor cell death, expression of proinflammatory cytokines, and glial responses, and preserves retinal structure and function. These results indicate that GA ameliorates RD by inhibiting inflammation in the blue LED-induced RD model and prove our hypothesis that HMGB1 can be a critical molecular target for inhibiting RD. The role of HMGB1 in the pathogenesis of blue-LED induced RD and amelioration of RD by GA is illustrated in Figure 8.

In the present study, the HMGB1 level was decreased in control RD mice, compared to that in normal control mice with restored HMGB1 in GA-treated RD mice. This result appears to be different from previous mentioned results showing increased HMGB1 in a rat retinal detachment model and in human eyes with retinal detachment (Arimura et al., 2009) as well as in dsRNA-induced RD model (Murakami et al., 2014). However, this discrepancy might be caused by the difference in the samples used for measuring the HMGB1 expression level, i.e., the two previous studies measured HMGB1 (released from photoreceptors and accumulated) in the vitreous (Arimura et al., 2009; Murakami et al., 2014), whereas the present study measured it directly in the retina. This explanation is corroborated by two previous reports, one mentioning that *HMGB1* is downregulated in a light-induced RD model (Krishnan et al., 2008) and another that HMGB1 immunohistochemistry shows decreased/absent staining in the ONL in an AAG-dependent alkylation-induced RD model (Allocca et al., 2019). Our immunohistochemical results also clearly demonstrate decreased HMGB1 in photoreceptors in the blue LED-induced RD model.

Lastly, we suggest that HMGB1 is a key molecule for photoreceptor survival in RD. This proposal is also illustrated in Figure 8 and derived from the following basis. First, although HMGB1 as a representative DAMP is suggested to trigger inflammation and aggravate RD (Arimura et al., 2009; Murakami et al., 2014; Allocca et al., 2019), there has been no direct evidence to prove this hypothesis. In addition, HMGB1 injection into the vitreous induces ganglion cell loss in the ganglion cell layer, but little photoreceptor loss in the ONL (Sakamoto et al., 2015). Second, although almost all photoreceptors in control RD retinas lose HMGB1 immunoreactivity, most of them look normal, not necrotic, based on the DAPI staining results in this study and the EM findings in our previous study (Kim G.H. et al., 2016). The same finding was reported in an AAG-dependent alkylation-induced RD model (Allocca et al., 2019). These findings do not match our general knowledge that HMGB1 is passively released from necrotic cells (Scaffidi et al., 2002; Lotze and Tracey, 2005; Klune et al., 2008). Taken together, we argue that HMGB1 may be a key survival factor for photoreceptors, as proposed in several pathological systems, including the heart (Funayama et al., 2013) and brain (Qi et al., 2007; Enokido et al., 2008). Considering that GA binds to HMGB1 that is bound to DNA and inhibits HMGB1 release from apoptotic chromatin (Mollica et al., 2007), and that HMGB1 functions in nuclear structure to regulate gene expression and DNA repair (Lange et al., 2008; Kang et al., 2014), GA administered before RD induction may bind the photoreceptor DNA and stabilize the genome against injury to photoreceptors or inhibit the expression of death-related genes such as caspase-3 and RIP1/RIP3 kinases. To confirm this point, we might need to evaluate retinal function in an RD model with photoreceptor-specific HMGB1 knockout mice in a future study.

## Conclusion

The present study reveals that GA inhibits the expression of proinflammatory cytokines, chemokines, and other inflammatory mediators in the retina *via* released HMGB1 inhibition, and additionally suppresses the expression of death-related genes in photoreceptors *via* binding to nuclear HMGB1, and thus, prevents RD progression and preserves retinal structure and function. These results suggest that HMGB1 is a key molecule in RD pathogenesis and a potent molecular target for the inhibition of RD, and that GA is a potential medication for the prevention or treatment of RD.

## Data Availability

The raw data supporting the conclusions of this manuscript will be made available by the authors, without undue reservation, to any qualified researcher.

## Ethics Statement

All mice-related experiments were handled according to the regulations of the Catholic Ethics Committee of the Catholic University of Korea, Seoul, which conform to the National Institute of Health (NIH) guidelines for the Care and Use of Laboratory Animals (NIH Publication No. 80-23), as revised in 1996. Experimental procedures were approved by the Institutional Animal Care and Use Committee at the College of Medicine, The Catholic University of Korea (Approval Numbers: CUMC 2016-0172-12 and 2017-0241-02).

## Author Contributions

I-BK conceived and designed the experiments. GK and S-SP set up the RD mouse model, performed ERG, TUNEL staining, and immunohistochemistry. YP contributed to taking confocal images. HK performed western blotting and densitometry. GK, S-SP, and I-BK wrote the manuscript. All authors read and approved the final manuscript.

## Conflict of Interest Statement

The authors declare that the research was conducted in the absence of any commercial or financial relationships that could be construed as a potential conflict of interest.

## References

[B1] Abu El-Asrar A. M. Siddiquei M. M. Nawaz M. I. Geboes K. Mohammad G. (2014). The proinflammatory cytokine high-mobility group box-1 mediates retinal neuropathy induced by diabetes. *Med. Inflamm.* 2014:10. 10.1155/2014/746415 24733965 PMC3964896

[B2] Allocca M. Corrigan J. J. Mazumder A. Fake K. R. Samson L. D. (2019). Inflammation, necrosis, and the kinase RIP3 are key mediators of AAG-dependent alkylation-induced retinal degeneration. *Sci. Signal.* 12:eaau9216. 10.1126/scisignal.aau9216 30755477 PMC7150588

[B3] Arimura N. Ki-I Y. Hashiguchi T. Kawahara K. Biswas K. K. Nakamura M. (2009). Intraocular expression and release of high-mobility group box 1 protein in retinal detachment. *Lab. Invest.* 89 278–289. 10.1038/labinvest.2008.165 19139725

[B4] Arroyo J. G. Yang L. Bula D. Chen D. F. (2005). Photoreceptor apoptosis in human retinal detachment. *Am. J. Ophthalmol.* 139 605–610. 10.1016/j.ajo.2004.11.046 15808154

[B5] Bandello F. Sacconi R. Querques L. Corbelli E. Cicinelli M. V. Querques G. (2017). Recent advances in the management of dry age-related macular degeneration: a review. *F1000Res.* 6:245. 10.12688/f1000research.10664.1 28529701 PMC5428517

[B6] Becerra E. M. Morescalchi F. Gandolfo F. Danzi P. Nascimbeni G. Arcidiacono B. (2011). Clinical evidence of intravitreal triamcinolone acetonide in the management of age-related macular degeneration. *Curr. Drug Targets* 12 149–172. 10.2174/138945011794182746 20887246

[B7] Chang S. W. Kim H. I. Kim G. H. Park S. J. Kim I. B. (2016). Increased expression of osteopontin in retinal degeneration induced by blue light-emitting diode exposure in mice. *Front. Mol. Neurosci.* 9:58. 10.3389/fnmol.2016.00058 27504084 PMC4958628

[B8] Chen X. L. Zhang X. D. Li Y. Y. Chen X. M. Tang D. R. Ran R. J. (2013). Involvement of HMGB1 mediated signalling pathway in diabetic retinopathy: evidence from type 2 diabetic rats and ARPE-19 cells under diabetic condition. *Br. J. Ophthalmol.* 97 1598–1603. 10.1136/bjophthalmol-2013-303736 24133029

[B9] Chinskey N. D. Besirli C. G. Zacks D. N. (2014). Retinal cell death and current strategies in retinal neuroprotection. *Curr. Opin. Ophthalmol.* 25 228–233 10.1097/icu.0000000000000043 24614145

[B10] Coleman H. R. Chan C. C. Ferris F. L. I. I. I. Chew E. Y. (2008). Age-related macular degeneration. *Lancet* 372 1835–1845. 10.1016/S0140-6736(08)61759-6 19027484 PMC2603424

[B11] Cook B. Lewis G. P. Fisher S. K. Adler R. (1995). Apoptotic photoreceptor degeneration in experimental retinal detachment. *Invest. Ophthalmol. Vis. Sci.* 36 990–996. 7730033

[B12] Cottet S. Schorderet D. F. (2009). Mechanisms of apoptosis in retinitis pigmentosa. *Curr. Mol. Med.* 9 375–383. 10.2174/156652409787847155 19355918

[B13] Ding X. Patel M. Chan C. C. (2009). Molecular pathology of age-related macular degeneration. *Prog. Retin. Eye Res.* 28 1–18. 10.1016/j.preteyeres.2008.10.001 19026761 PMC2715284

[B14] Donovan M. Cotter T. G. (2002). Caspase-independent photoreceptor apoptosis in vivo and differential expression of apoptotic protease activating factor-1 and caspase-3 during retinal development. *Cell Death Differ.* 9 1220–1231. 10.1038/sj.cdd.4401105 12404121

[B15] Dunaief J. L. Dentchev T. Ying G. S. Milam A. H. (2002). The role of apoptosis in age-related macular degeneration. *Arch. Ophthalmol.* 120 1435–1442. 12427055 10.1001/archopht.120.11.1435

[B16] Dvoriantchikova G. Hernandez E. Grant J. Santos A. R. Yang H. Ivanov D. (2011). The high-mobility group box-1 nuclear factor mediates retinal injury after ischemia reperfusion. *Invest. Ophthalmol. Vis. Sci.* 52 7187–7194. 10.1167/iovs.11-7793 21828158 PMC3207720

[B17] Enokido Y. Yoshitake A. Ito H. Okazawa H. (2008). Age-dependent change of HMGB1 and DNA double-strand break accumulation in mouse brain. *Biochem. Biophys. Res. Commun.* 376 128–133. 10.1016/j.bbrc.2008.08.108 18762169

[B18] Funayama A. Shishido T. Netsu S. Narumi T. Kadowaki S. Takahashi H. (2013). Cardiac nuclear high mobility group box 1 prevents the development of cardiac hypertrophy and heart failure. *Cardiovasc. Res.* 99 657–664. 10.1093/cvr/cvt128 23708738 PMC3746952

[B19] Gregory-Evans K. Bhattacharya S. S. (1998). Genetic blindness: current concepts in the pathogenesis of human outer retinal dystrophies. *Trends Genet.* 14 103–108. 10.1016/s0168-9525(98)01402-4 9540407

[B20] Guadagni V. Novelli E. Piano I. Gargini C. Strettoi E. (2015). Pharmacological approaches to retinitis pigmentosa: a laboratory perspective. *Prog. Retin. Eye Res.* 48 62–81. 10.1016/j.preteyeres.2015.06.005 26113212

[B21] Hoppe G. Rayborn M. E. Sears J. E. (2007). Diurnal rhythm of the chromatin protein Hmgb1 in rat photoreceptors is under circadian regulation. *J. Comp. Neurol.* 501 219–230. 10.1002/cne.21248 17226794

[B22] Kang R. Chen R. Zhang Q. Hou W. Wu S. Cao L. (2014). HMGB1 in health and disease. *Mol. Aspects Med.* 40 1–116. 10.1016/j.mam.2014.05.001 25010388 PMC4254084

[B23] Kao T. C. Wu C. H. Yen G. C. (2014). Bioactivity and potential health benefits of licorice. *J. Agric. Food Chem.* 62 542–553. 10.1021/jf404939f 24377378

[B24] Karlstetter M. Ebert S. Langmann T. (2010). Microglia in the healthy and degenerating retina: insights from novel mouse models. *Immunobiology* 215 685–691. 10.1016/j.imbio.2010.05.010 20573418

[B25] Kauppinen A. Paterno J. J. Blasiak J. Salminen A. Kaarniranta K. (2016). Inflammation and its role in age-related macular degeneration. *Cell. Mol. Life Sci.* 73 1765–1786. 10.1007/s00018-016-2147-8 26852158 PMC4819943

[B26] Kim G. H. Kim H. I. Paik S. S. Jung S. W. Kang S. Kim I. B. (2016). Functional and morphological evaluation of blue light-emitting diode-induced retinal degeneration in mice. *Graefes Arch. Clin. Exp. Ophthalmol.* 254 705–716. 10.1007/s00417-015-3258-x 26743754

[B27] Kim H. I. Paik S. S. Kim G. H. Kim M. Kim S. H. Kim I. B. (2016). Neuroprotective effect of NecroX-5 against retinal degeneration in rodents. *Neuroreport* 27 1128–1133. 10.1097/WNR.0000000000000666 27541272

[B28] Kim S. W. Jin Y. Shin J. H. Kim I. D. Lee H. K. Park S. (2012). Glycyrrhizic acid affords robust neuroprotection in the postischemic brain via anti-inflammatory effect by inhibiting HMGB1 phosphorylation and secretion. *Neurobiol. Dis.* 46 147–156. 10.1016/j.nbd.2011.12.056 22266336

[B29] Klune J. R. Dhupar R. Cardinal J. Billiar T. R. Tsung A. (2008). HMGB1: endogenous danger signaling. *Mol. Med.* 14 476–484. 10.2119/2008-00034.Klune 18431461 PMC2323334

[B30] Krishnan J. Chen J. Shin K. J. Hwang J. I. Han S. U. Lee G. (2008). Gene expression profiling of light-induced retinal degeneration in phototransduction gene knockout mice. *Exp. Mol. Med.* 40 495–504. 18985007 10.3858/emm.2008.40.5.495 PMC2679354

[B31] Lange S. S. Mitchell D. L. Vasquez K. M. (2008). High mobility group protein B1 enhances DNA repair and chromatin modification after DNA damage. *Proc. Natl. Acad. Sci. U.S.A.* 105 10320–10325. 10.1073/pnas.0803181105 18650382 PMC2492475

[B32] Lin W. Xu G. (2019). Autophagy: a role in the apoptosis, survival, inflammation, and development of the retina. *Ophthalmic Res.* 61 65–72. 10.1159/000487486 29694961

[B33] Liu L. Jiang Y. Steinle J. J. (2017). Inhibition of HMGB1 protects the retina from ischemia-reperfusion, as well as reduces insulin resistance proteins. *PLoS One* 12:e0178236. 10.1371/journal.pone.0178236 28542588 PMC5441648

[B34] Lotze M. T. Tracey K. J. (2005). High-mobility group box 1 protein (HMGB1): nuclear weapon in the immune arsenal. *Nat. Rev. Immunol.* 5 331–342. 10.1038/nri1594 15803152

[B35] Madeira M. H. Boia R. Santos P. F. Ambrósio A. F. Santiago A. R. (2015). Contribution of microglia-mediated neuroinflammation to retinal degenerative diseases. *Med. Inflamm.* 2015:673090. 10.1155/2015/673090 25873768 PMC4385698

[B36] McMurtrey J. J. Tso M. O. M. (2018). A review of the immunologic findings observed in retinitis pigmentosa. *Surv. Ophthalmol.* 63 769–781. 10.1016/j.survophthal.2018.03.002 29551596

[B37] Ming L. J. Yin A. C. (2013). Therapeutic effects of glycyrrhizic acid. *Nat. Prod. Commun.* 8 415–418. 23678825

[B38] Mollica L. De Marchis F. Spitaleri A. Dallacosta C. Pennacchini D. Zamai M. (2007). Glycyrrhizin binds to high-mobility group box 1 protein and inhibits its cytokine activities. *Chem. Biol.* 14 431–441. 10.1016/j.chembiol.2007.03.007 17462578

[B39] Murakami Y. Matsumoto H. Roh M. Giani A. Kataoka K. Morizane Y. (2014). Programmed necrosis, not apoptosis, is a key mediator of cell loss and DAMP-mediated inflammation in dsRNA-induced retinal degeneration. *Cell Death Differ.* 21 270–277. 10.1038/cdd.2013.109 23954861 PMC3890945

[B40] Murakami Y. Matsumoto H. Roh M. Suzuki J. Hisatomi T. Ikeda Y. (2012). Receptor interacting protein kinase mediates necrotic cone but not rod cell death in a mouse model of inherited degeneration. *Proc. Natl. Acad. Sci. U.S.A.* 109 14598–14603. 10.1073/pnas.1206937109 22908283 PMC3437885

[B41] Murakami Y. Notomi S. Hisatomi T. Nakazawa T. Ishibashi T. Miller J. W. (2013). Photoreceptor cell death and rescue in retinal detachment and degenerations. *Prog. Retin. Eye Res.* 37 114–140. 10.1016/j.preteyeres.2013.08.001 23994436 PMC3871865

[B42] Nita M. Grzybowski A. Ascaso F. J. Huerva V. (2014). Age-related macular degeneration in the aspect of chronic low-grade inflammation (pathophysiological parainflammation). *Med. Inflamm.* 2014:930671. 10.1155/2014/930671 25214719 PMC4152952

[B43] Papermaster D. S. Windle J. (1995). Death at an early age. Apoptosis in inherited retinal degenerations. *Invest. Ophthalmol. Vis. Sci.* 36 977–983. 7730031

[B44] Portera-Cailliau C. Sung C. H. Nathans J. Adler R. (1994). Apoptotic photoreceptor cell death in mouse models of retinitis pigmentosa. *Proc. Natl. Acad. Sci. U.S.A.* 91 974–978. 10.1073/pnas.91.3.974 8302876 PMC521436

[B45] Qi M. L. Tagawa K. Enokido Y. Yoshimura N. Wada Y. Watase K. (2007). Proteome analysis of soluble nuclear proteins reveals that HMGB1/2 suppress genotoxic stress in polyglutamine diseases. *Nat. Cell Biol.* 9 402–414. 10.1038/ncb1553 17384639

[B46] Sakamoto K. Mizuta A. Fujimura K. Kurauchi Y. Mori A. Nakahara T. (2015). High-mobility group box-1 is involved in NMDA-induced retinal injury the in rat retina. *Exp. Eye Res.* 137 63–70. 10.1016/j.exer.2015.06.003 26079740

[B47] Sakamoto K. Okuwaki T. Ushikubo H. Mori A. Nakahara T. Ishii K. (2017). Activation inhibitors of nuclear factor kappa B protect neurons against the NMDA-induced damage in the rat retina. *J. Pharmacol. Sci.* 135 72–80. 10.1016/j.jphs.2017.09.031 29110956

[B48] Sancho-Pelluz J. Arango-Gonzalez S. Kustermann F. J. Romero T. van Veen E. Zrenner P. (2008). Photoreceptor cell death mechanisms in inherited retinal degeneration. *Mol. Neurobiol.* 38 253–269. 10.1007/s12035-008-8045-9 18982459

[B49] Sato K. Li S. Gordon W. C. He J. Liou G. I. Hill J. M. (2013). Receptor interacting protein kinase-mediated necrosis contributes to cone and rod photoreceptor degeneration in the retina lacking interphotoreceptor retinoid-binding protein. *J. Neurosci.* 33 17458–17468. 10.1523/JNEUROSCI.1380-13.2013 24174679 PMC3812510

[B50] Scaffidi P. Misteli T. Bianchi M. E. (2002). Release of chromatin protein HMGB1 by necrotic cells triggers inflammation. *Nature* 418 191–195. 10.1038/nature00858 12110890

[B51] Shen L. Cui Z. Lin Y. Wang S. Zheng D. Tan Q. (2015). Anti-inflammative effect of glycyrrhizin on rat thermal injury via inhibition of high-mobility group box 1 protein. *Burns* 41 372–378. 10.1016/j.burns.2014.05.008 25440843

[B52] Trichonas G. Murakami Y. Thanos A. Morizane Y. Kayama M. Debouck C. M. (2010). Receptor interacting protein kinases mediate retinal detachment-induced photoreceptor necrosis and compensate for inhibition of apoptosis. *Proc. Natl. Acad. Sci. U.S.A.* 107 21695–21700. 10.1073/pnas.1009179107 21098270 PMC3003048

[B53] Viringipurampeer I. A. Bashar A. E. Gregory-Evans C. Y. Moritz O. L. Gregory-Evans K. (2013). Targeting inflammation in emerging therapies for genetic retinal disease. *Int. J. Inflam.* 2013:581751. 10.1155/2013/581751 23509666 PMC3594980

[B54] Wubben T. J. Besirli C. G. Zacks D. N. (2016). Pharmacotherapies for retinal detachment. *Ophthalmology* 123 1553–1562. 10.1016/j.ophtha.2016.02.040 27040150

[B55] Xu G. Z. Li W. W. Tso M. O. (1996). Apoptosis in human retinal degenerations. *Trans. Am. Ophthalmol. Soc.* 94 411–430. 8981707 PMC1312106

[B56] Yoshida N. Ikeda Y. Notomi S. Ishikawa K. Murakami Y. Hisatomi T. (2013). Clinical evidence of sustained chronic inflammatory reaction in retinitis pigmentosa. *Ophthalmology* 120 100–105. 10.1016/j.ophtha.2012.07.006 22986109

